# Improving forensic healthcare: ARMED, a new telemedical examination

**DOI:** 10.1007/s00414-025-03463-9

**Published:** 2025-03-03

**Authors:** Yasmeen M. Taalab, Dorothea Kaufmann, Aysche Landmann, Emily Marie Ungermann, Sarah Heinze, Barbara Stöttner, Anastasia Tsaklakidis, Andreas Schroff, Florian Konrad, Alexander Mezger, Sophia Schlenzig, Robert Yen, Kathrin Yen

**Affiliations:** 1 Institute of Forensic and Traffic Medicine, University Hospital Heidelberg, Heidelberg, Germany; 2 Medical University Graz, Institute of Forensic Medicine, Graz, Austria; 3 Ludwig Maximilian University of Munich, Institute of Forensic Medicine, Munich, Germany; 4 Independent Researcher, Heidelberg, Germany; 5 Pediatrics Unit, Ortenau Hospital Offenburg-Kehl, Kehl, Germany; 6 Pediatrics Unit, SLK-Hospitals Heilbronn GmbH, Heilbronn, Germany; 7 Pediatrics Unit, Oberschwaben-Hospital GmbH Ravensburg, Ravensburg, Germany; 8 Independent Researcher, Mannheim, Germany

**Keywords:** ARMED, Telemedicine, Violence victims, Forensic documentation, Augmented reality, Assisted reality

## Abstract

**Background:**

the Istanbul Convention demands care to victims of violence while upholding forensic standards. Victims, however, often seek medical help at hospitals where the availability of forensic experts is limited. This results in overlooked injuries and lost or damaged evidence, ultimately impacting court proceedings and identification of individuals at risk. The aim of this paper was to establish real-time remote guidance for distant physicians during the forensic examination of violence victims.

**Methods:**

Augmented Reality Assisted Medical Evidence Collection and Documentation (ARMED) was established in Heidelberg at the Institute for Forensic and Traffic Medicine (IFTM) in 2023 as an innovative telementoring model. Video-teleconferencing components including a head-mounted device (HMD), a customized software package, hardware devices, and a data management portal were employed to facilitate seamless expert care delivery, data sharing, and to ensure privacy and confidentiality. ARMED platform was evaluated in three partner hospitals with parameters including internet connection stability, clarity of live-streaming and audio-visual communication, the quality of photos, and the safety of data management.

**Results:**

The combination of RealWear Navigator 500 as HMD, a customized version of videoconferencing software, and a portal server system for safe and secure patient data management constituted a robust, user-friendly, and practical telemedicine solution.

**Conclusion:**

ARMED facilitates real-time communication between healthcare providers and forensic experts, enhancing their ability to recognize and detect injuries effectively. This holds the potential to significantly improve the process of evidence collection for documenting cases of violence, ultimately aiding in the pursuit of justice and the protection of victims.

## Introduction

Violence has detrimental long-term health and social consequences, which can even lead to fatalities. However, women, children and elderly are frequently affected by non-fatal physical, sexual and psychological violence [1]. In Germany, the annually released Police Crime Statistics registered 197,202 cases of violent crimes in 2022. Amongst these, 3,516 cases of physical and mental child abuse, and 15,520 cases of child sexual abuse were reported. These numbers, however, refer only to cases reported to the police, while the overall number of unreported crimes is likely immensely higher [2]. An obligation to enhance the protection of victims arises from the Istanbul Convention, an international agreement that came into force in Germany in 2018 [3]. Accordingly, several measures have been initiated including the establishment of new legal foundations aimed at improving forensic medical care and facilitating access to independent clinical forensic examinations. According to the German Social Code (Sozialgesetzbuch; SGB V § 27) and without prior involvement of the police (“procedure independent”), health-insured individuals have the right to get confidential evidence collection of their bodies, including injury documentation, laboratory examinations, and proper storage of secured findings in cases where health harms resulting from any kind of violence were indicated. Additionally, statutory health insurances are obligated to cover the associated costs (SGB V § 132k) [4, 5].

Forensic medical care for victims of violence is currently however inadequate. Even in larger metropolitan areas, there is insufficient 24/7 availability of forensic experts to take responsibility for evidence collection, securing, and documentation for cases not officially reported to the authorities (commonly known as “non-procedural” cases). Facing the absence of a procedure-independent service, this results in a considerable loss of information [1, 6, 7].

Moreover, victims primarily seek assistance at general hospitals where there is also shortage of forensic experts. Consequently, examinations might be performed by physicians lacking expertise in forensic medicine. A statistically significant difference was detected in the quality of clinical forensic examinations conducted by clinicians compared to forensic examiners regarding forensically relevant aspects, such as full-body examination, qualified photo documentation, and sufficient information on injuries [1]. Therefore, examination needs to be carried out by forensic experts, not only to guarantee that the documentations are adequate and admissible as court evidence, but also to identify persons at risk [8–10].

In summary, there is an urgent need to improve accessibility to forensic experts without compromising the quality of care [7]. Telemedicine serves as a valuable tool to bridge this gap, ensuring that victims of violence receive forensic evaluation even in locations with restricted access to forensic expertise. Telemedicine has grown significantly over the past few decades, particularly during the COVID-19 pandemic [11–14], and even in the post-pandemic era [15–17]. Many applications in medically underserved regions [18], and pilot projects in various specialized fields have been reported [19–21]. However, there is no previously documented telemedicine system facilitating real-time remote guidance by a forensic expert to distant physicians when examining victims of violence.

In 2020, ARMED, an acronym standing for Augmented Reality Assisted Medical Evidence Collection and Documentation, was launched at the Institute of Forensic and Traffic Medicine (IFTM) of the Heidelberg University Hospital (Universitätsklinikum Heidelberg, UKHD), funded by the Ministry of Social Affairs, Health, and Integration and co-financed by state funds approved by the Baden-Wuerttemberg state parliament. Its primary goal was to plan, develop, evaluate, and pilot test a telementoring platform for joint clinical-forensic examinations, enabling physicians at remote locations to conduct guided forensic examinations of children who were reported as possible victims of violence. In the near future, the scope shall be extended to include victims of violence of all ages.

This article provides an in-depth description of the ARMED design, system requirements, digital components, and workflow. The significance as well as limitations associated with the adoption of ARMED in regular practice will be addressed additionally.

## Methods

The ARMED platform was developed in 4 steps: (1) analysis, (2) design, (3) implementation, and (4) evaluation and pilot system testing.

### Analysis: determination and definition of the system and user requirements

Three brainstorming workshops were conducted to gather general prerequisites, personal roles, activities, and tools that mediate the activities. At the outset, we identified the key roles involved in the process of seeking and providing care for victims of violence. The detailed description of the roles, the resources they require, and the challenges are illustrated in Table 1.

**Table 1:** System and user requirements, personal roles, and prerequisites to build-up the ARMED platform

Personal Roles		Description
Forensic expert		Professional forensic physician at IFTM
Forensic supervisor		Experienced forensic consultant (e.g. senior physician) at IFTM who can access the examination whenever needed as a third participant during the telemedical assessment.
Physician		Non-forensic physician conducting the clinical forensic examination to the victim on-site
Victim of violence		Target group of 0-18-years-old children suffering various types of violence.
Operator		Responsible for ensuring the functionality of the system, providing support to users, promptly address any errors, and arrange for replacement devices whenever needed during the procedure.

General Requirements for all Users		Description
Users		• Basic knowledge of forensic medical examination process, emergency psychology, physician-patient communication post-violence, and telemedicine with traumatized children. • Familiarity with equipment and interdisciplinary examinations under supervision. • Willingness to use telemedicine apps, engage in evaluations, and embrace quality improvement. • Completed training provided by IFTM, including telemedicine and forensic examination techniques, with ongoing instruction as required.

Requirements with respect to the System		Description
General requirements	Functional requirements	• Data protection compliant with medical standards and legal requirements according to German General Data Protection Regulation (GDPR). • Intuitive Graphical user interfaces (GUIs) for user-friendly experience. • Central storage of data, centralized long-term storage. • Visible overview of case data for all users. • Document edits with timestamps and user info. • Management of users with varying authorizations. • Add users with master data and center assignment. • Management of examination centers and user permissions. • Implementation of automated data deletion procedures. • Secure, unmodifiable, signed image findings. • Interface with IFTM Heidelberg’s patient data management system i/med (Dorner Healthcare IT Solutions, Germany). • Display of video, case data, and dialog in separate adjustable windows. • External site’s forensic exam must match IFTM’s high quality standards. • Telemedicine app aids external physician. • Remote experts virtually assist in real time.
	Non-functional requirements and prerequisites	• Standard hardware components. • High-resolution images, videos clearly depicting even very small findings. • True-to-color camera and screen displays. • Connection via WLAN, 5G, or broadband LAN. • Customizable software.
Requirements concerning the users	Functional requirements of the system for the physician	• Input of general case data: patient, guardian, admission, contact information. • Recording of case examination data, including start time. • Recording of examiner details, procedure history, findings. • Photo and video documentation, documentation of evidence collection. • Communication between physician and expert. • Sharing of real-time video of physical exam. • Communication, exam stored as audio-video if needed. • Possibility of multiple parallel workflows and chaining. • Access to radiological images via PACS system. • Written feedback and evaluation after examination. • Inclusion of supervisor if needed. • System displays real-time graphical guidance to examiner during examination using augmented reality (AR). • System guides examiner through configurable graphical workflow in examination dialogue (AR). • System provides graphical help for radiological images on tablet PC or screen. • Examiner can annotate images using stylus on tablet or screen.
	Functional requirements of the system for the forensic expert	• Same point of view as physician during examination. • Audio communication with physician. • Saving of audio-video remotely. • Record of feedback and evaluation before completion. • Inclusion of supervisor if needed. • Saving of diagnostic and radiological images with graphical guidance, linking to the examination • Possibility to document the findings parallel to the examination
	Functional requirements of the system for the supervisor	• Selection of case and ongoing examination from own list. • Same requirements as expert, but supervisory role. • Assessment of quality statistically, by evaluations. • Can view feedback for investigators/companions. • Evaluation of examination counts statistically.

### System design and development

This phase was focused on the conceptualization of previously defined requirements and the construction of a prototype featuring essential interfaces for users. Special emphasis has been set on data protection and privacy.

**Fig. 1:**
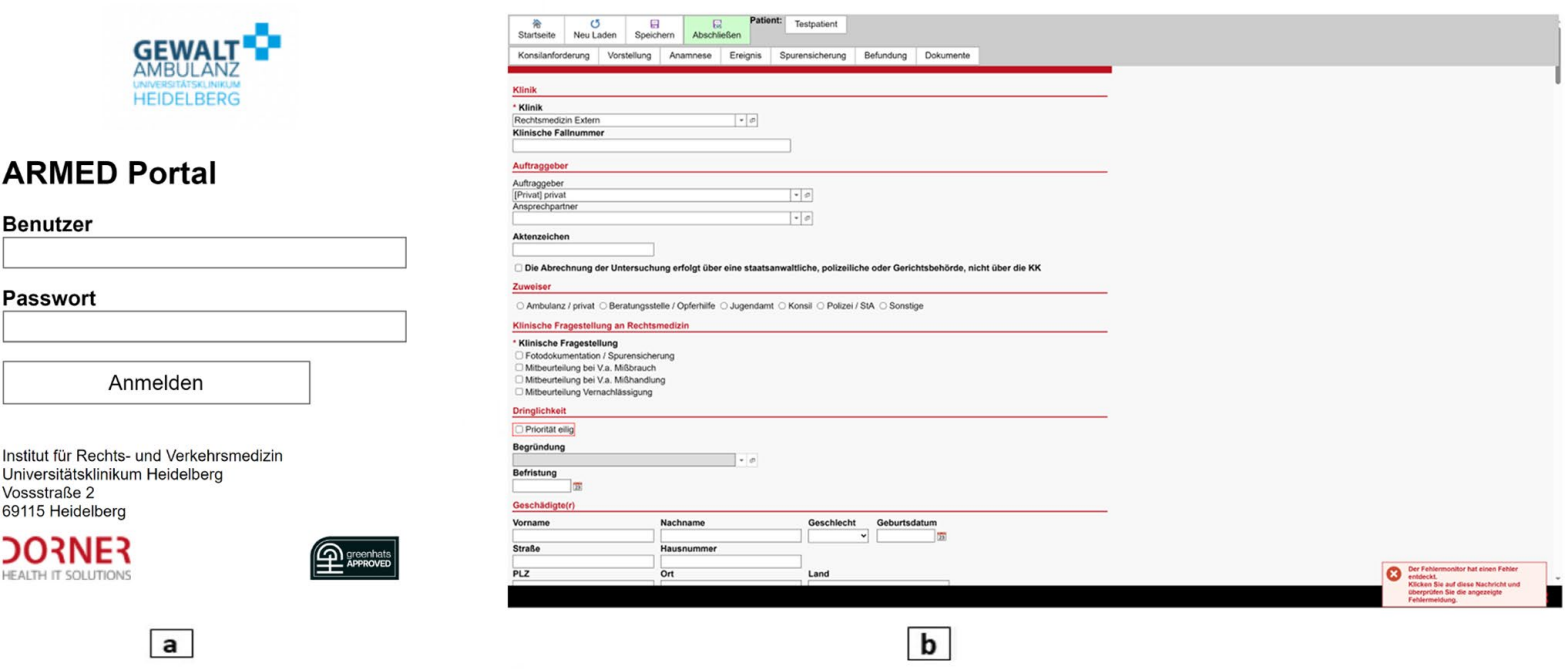
ARMED portal server system. (**a**) the login page. (**b**) The starting page showing the forms for data entry are illustrated. The collected data encompasses the following categories: personal contact details such as name, first name, address, phone number, and email address. Additionally, personal information like age, gender, and birthdate is recorded. Administrative data pertains to insurance information. Special data covers health-related information

Four main basic steps were considered in building the prototype: (1) An onsite physician encounters a victim of violence or suspected violence. (2) The physician connects with a remote forensic expert using an HMD and simultaneously accesses a portal server to enter personal data, incident information, and detected injuries. (3) The forensic expert utilizes a hardware device with a customized software package to follow the onsite physician and accesses the portal server system to review or supplement the entered data or to document findings in parallel with the examination. (4) The expert can remotely guide the physician throughout the entire examination process via a real-time video teleconferencing tool. Accordingly, five specific artifacts (i.e., software and hardware devices) were identified and set up:

- The “portal server system” reflecting a data management system designed and developed in collaboration with Dorner Health IT Solutions to enable the entry, saving, and digital transfer of personal and incident-related data to the electronic case file system named i/med at the IFTM in Heidelberg. It operates entirely through a web-based interface, where users log in with their username and password. Physicians are able to create new case profiles, save orders temporarily for later additions, or complete them immediately. Users can also view a list of all the orders processed on the start page. Both physicians and experts have simultaneous access to the case file, facilitating data sha (Fig. 1).
- i/med by Dorner Health IT Solutions is the electronic case file system of IFTM which is compatible with the portal server system. Case files entered into the portal server system, along with photos, radiological images, and all other related documents, are transferred directly to i/med for further interpretation and archiving. The data is securely saved on UKHD archive servers, utilizing SSL end-to-end encryption for data transmission.
- An HMD device, preferentially offering XR solutions, particularly augmented reality (AR)/mixed reality (MR) solutions to be worn by the physician to facilitate the communication with the forensic expert.
- Off-the-shelf hardware (Microsoft surface) is connected through WIFI or a 5G communication network to receive the video feed from the physician. Three additional monitors were used at the expert workplace to view case file, documents, and any extra materials e.g. radiological images.
- A customized software package was installed on the devices used at both workplaces. It serves to display information and facilitate task navigation during the examination. It allows to invite more participants to the call session whenever needed, for example, senior forensic consultants (“supervisor”), psychiatrists, or radiologists. They can also provide instructions or recommendations to the examining physician.

**Fig. 2:**
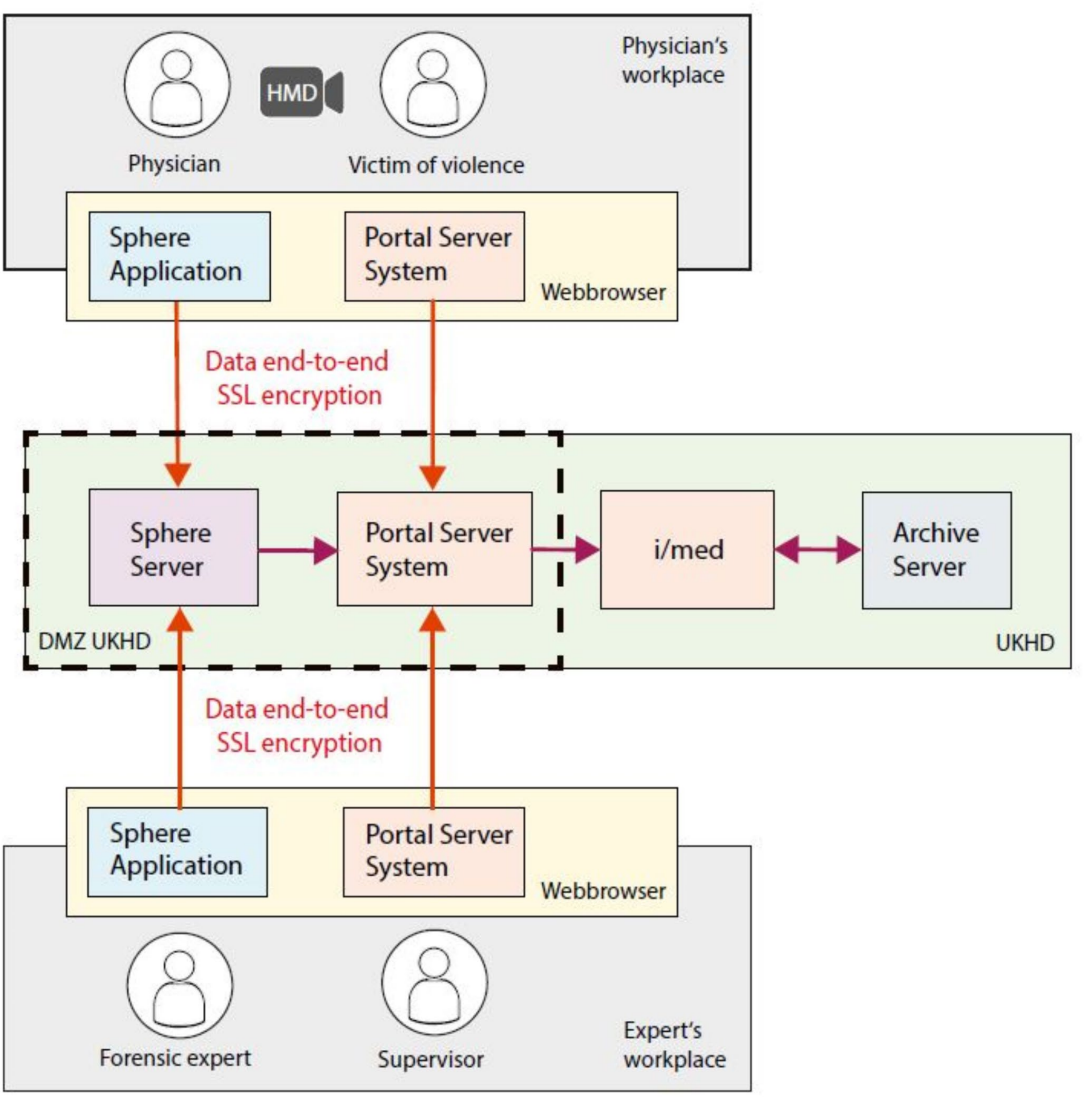
The architecture of the ARMED platform illustrating its structural components (organization, subjects, artifacts, context, and product). Articulation components and interfaces working together offering access to high-quality care for victim after violence

The detailed architecture of the ARMED platform illustrating its structural components is shown in Fig. 2.

Several enhancements were accomplished to improve the portal server system to manage the data entry, upload documents and photos, and portal responsiveness on different devices. Furthermore, the software Sphere© was customized throughout development of the ARMED system. The developed features include:

- Customizations around the on-premises setup of the UKHD (containerized deployment / on-premises settings shortcut / user authentication improvements).
- Organization of on-the-fly attachments by both user and date within the “user created media” folder in the Sphere^®^ portal management.
- Implementation & optimization of HQ screenshot functionality, screensharing functionality, and quality of the streaming service the between PC and RWN500.
- Support for conference calling functionalities on RWN500.
- Support for both landscape modes on RWN500 to allow wearing it on both eyes.
- Extension of the recording functionality on PC to the entire UI and not only the incoming video feed.

Developed, but not used at this point:

- Extension of network features to collaboratively execute workflows during remote assistance sessions.
- Implementation of loop functionality to efficiently register findings through pre-defined workflows.
- Implementation of voice-to-text functionality to efficiently register findings through workflows.
- Implementation of timer & preview functionality for pictures captured on HL2^TM^.
- Implementation of HQ screenshot functionality between PC and HL2^TM^.

The designed layout comprises of three monitors and additional off-the-shelf hardware connected through WIFI or 5G communication network at the “expert” workplace. The physician at the distant workplace wears the HMD whilst conducting the victim examination. The examination is captured by the headtracking camera. The HMD unit microphone and headphones permit verbal communication with the expert, who looks at a large display screen, which displays the real-time image of the examination from the external physician’s HMD unit. The commercially available software Sphere© (Sphere, formerly HoloOne, Switzerland) was customized to match ARMED use case. It serves as a videoconferencing platform, enables task navigation, and allows to take photos during the examination.

ARMED initially adopted AR/MR solutions, thus the HoloLens 2 (HL2^TM^) was used as an untethered optically see-through HMD developed by Microsoft [22]. HL2^TM^ ran the Windows Mixed Reality platform under the Windows 10 operating system which enabled the user to obtain immediate insight into the examination workflow, directly overlaid within their view. A remote assistance functionality enabled HL2^TM^ users to communicate with colleagues working remotely on tablets or desktop PCs through the competency software package. It was able to provide graphical guidance to the physician using AR. Additionally, it provided access to radiological images via the PACS system. With the replacement of the HMD, the use of AR/MR has been postponed until the development of new, easy-to-use devices. Instead, an Assisted-Reality (aR) Device, the Realwear Navigator 500 (RWN500), was utilized.

**Fig. 3:**
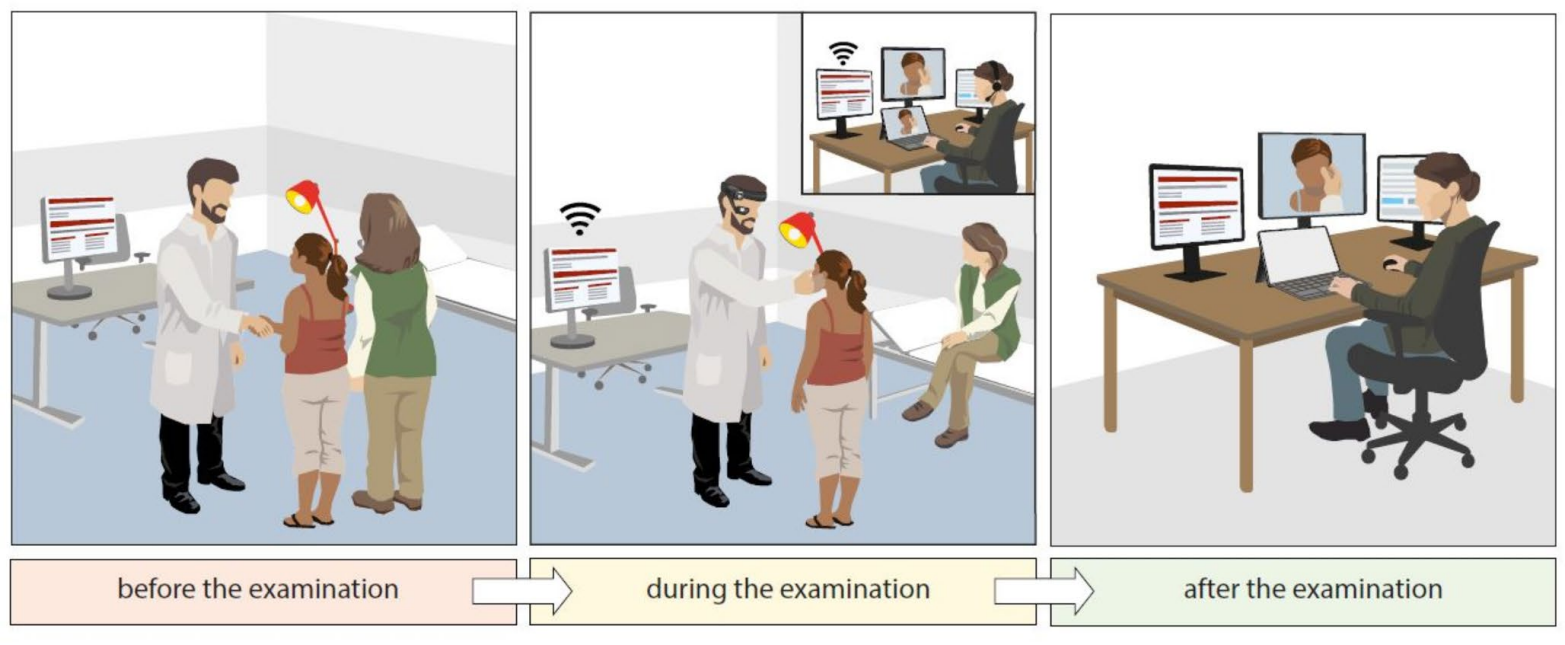
ARMED workflow. *Before the examination*: The physician at a distant location welcomes the victim of violence and the accompanying legal guardian(s). Prior to the examination, the entire procedure is explained to the victim and the legal guardian(s). The necessary documents and informed declaration of consent are signed. The initial information regarding personal data, incident details, medical history, and preliminary findings of the examination are entered into the portal server system. *During the examination*: To establish the real-time communication, the physician wears the HMD and connects to a remote forensic expert using customized Sphere©, where the examination is actively monitored by an expert on an off-the-shelf PC with a suitably large screen (no storage of video stream). The forensic expert sees exactly what the physician sees and guides the physician on how to proceed further. Evidence photos are taken during the examination and are added to the corresponding case file in the portal server system opened on the PC. When entering the master data of the victim, the portal server system generates a case ID, to which all data related to the case is then linked. Data protection is ensured throughout the whole procedure. *After the examination*: The physician debriefs the patient, with the forensic expert following the conversation to assist if necessary. Afterwards, findings, photos, and any additional documentary material (e.g., radiological findings) are exchanged between the non-forensic and forensic expert involved in the telecommunication session. The forensic expert then takes responsibility for evidence documentation and interpretation, which might eventually proceed to court

After defining the components, the set of steps to carry out an ARMED examination was developed as a “workflow” with three defined stages; before, during, and after the examination (Fig. 3). Checklists for user preparedness, workplace readiness, and the necessary documents were prepared.

During the examination process, the forensic expert will guide the remote physician through the entire procedure, including taking the victim’s history of the incident and conducting a detailed head-to-toe assessment to identify injuries or findings on the body. Injuries or findings will then be documented by taking photographs and measuring them using appropriate tools.

If trace evidence is found, the forensic expert will instruct the remote physician to follow specific steps for proper collection:

- Document and photograph the evidence.
- Secure the evidence by placing it in a paper bag or envelope.
- Seal the bag or envelope with tape, ensuring the examiner initials, dates, and marks the time across the sealed area.
- Label the container with the patient’s identifying details.
- Sign and date the envelope, with the examiner noting the time.

Biological evidence may include blood, skin, hair, semen, saliva, and urine.

- Swabs (cotton-tipped applicators) may be collected from various areas, such as the buccal cavity, oral cavity, skin, fingernails, bite marks, perineal, perianal, vaginal, cervical os, penile, scrotal, and rectal regions.
- Hair samples are stored in an envelope.
- The same procedures for packaging, securing, and preserving trace evidence apply to biological evidence.

Furthermore, clothing worn by an individual at the time of the incident often contains physical or biological evidence that must be preserved. If the victim is still wearing the clothes from the incident, all items should be considered evidence.

In addition to the system development, data protection plans and a data protection impact assessment according to German General Data Protection Regulation (GDPR) were filed. Photos taken during examinations are stored within the on-premise sphere management portal in which the data remain within the confines of the UKHD’s infrastructure and IT environment. Photos are downloaded from Sphere© management portal to IFTM’s local server, and uploaded to the portal server system and i/med.

### Implementation

This step was focused on creating a layout that includes all the navigation features for the ARMED components and workflow. Before implementing a real environment with real users and real cases, two things happened: (1) an end-to-end system technical testing were performed by system developers and internal users to evaluate the functional design to detect and correct errors, and to reduce technical risks. (2) Errors that have been identified were immediately corrected.

### System pilot testing

This phase was focused on testing the functional design according to the proposed structural design and on improvements that resulted from internal user feedback. The quality of ARMED was assessed by performing end-to-end system functional testing to ensure the robustness of the artifacts. The effectiveness was also evaluated from a human perspective through formative field-based usability testing to identify and reduce user-related risks or concerns. The piloting took place in the real environment of three partner hospitals by the real users working on fictional cases. Partner hospitals were chosen based on their location, annual number of patients and availability of 24-hour emergency care. SLK-Kliniken Heilbronn GmbH, Ortenau Klinikum gKAöR in Offenburg and Oberschwabenklinik gGmbH in Ravensburg matched the criteria and had no access to forensic experts on-site at that time.

Before launching ARMED into routine delivery, its usability and functionality were evaluated using various parameters. The testing parameters employed across the nine testing sessions, comprising three test sessions in each partner hospital, are summarized in Table 2. Scenario-based cases and simulated injuries were prepared prior to conducting the examinations. Additionally, questionnaires were developed to gather feedback on the examination process from the physician, the expert, and the legal guardian of the examined victim.

End-to-end system testing was conducted using HL2^TM^ and subsequently the RWN500 as an aR solution. Additionally, alongside ARMED-system testing, the quality of photos captured with the RWN500 using Sphere 3.0 was evaluated. For this purpose, various objects were photographed from different distances, and image quality was assessed based on visual and morphological criteria. To evaluate the quality of the ARMED video stream transmission, two forensic doctors in two separate settings were asked to evaluate the same injury. One expert directly examined the injury while wearing the HMD, and the other doctor viewed it via the ARMED video stream. A comparison of both descriptions was conducted, considering the type, size, color, and shape of all injuries.

**Table 2:** Testing parameters for the usability and functionality of ARMED used during system pilot testing

**Organizational regulation**
Commitment to telemedicine development efforts. Vision, priorities, and goals for implementing telemedicine.
**Technology: considering the Technical infrastructure needed for telemedicine in practice**
Internet for telecommunication connection • Internet availability • Internet connectivity quality • Internet bandwidth Equipment, software or services • HMDs ○ Interaction design Hands-free interaction Speech recognition and touch gestures ○ Information visualization Specific display device Amount of data displayed Legibility and the available field of view for perceiving surrounding information ○ Suitability for the context of use Weight Comfortability Care and Prepare-Principle • Software ○ User acceptance testing ○ Usability testing ○ Functional testing ○ Integration testing ○ End-to-End testing of the entire system ○ Performance testing ○ Compatibility testing ○ Need for extra aids e.g. headsets The involvement of the users in reviewing and selecting the technology Availability of IT staff or to provide technical expertise, technical support and troubleshooting.
**Physical space**
Designated space with an appropriate layout, privacy, and adequate lighting, and equipment
**Scheduling and workflow**
Administrative workflows: how the service fits within the daily workflow • Scheduling • Health insurance • Billing • Medical documentation and informed consent • Communication between staff Clinical workflows: Flow of the examination
**Photos documentation**
Photo quality • Sharpness • Contrast • Noise • Resolution • Color accuracy • Dynamic range
**Assessment Approach**
Success factors • Cost effectiveness • Staff engagement • Users/victim satisfaction • Improved outcomes Methods in place for soliciting feedback from providers, staff and victim/patients: prepared evaluation forms. Making improvements to services and administrative procedures based on feedback from providers, staff, and victims/patients.
**Privacy and security** Data protection impact assessment (Datenschutz-Folgenabschätzung (DSFA)) complies to article 35 GDPR (Datenschutzgrundverordnung (DSGVO)), German Institute for Standardization e.V. (Deutsches Institut für Normung e.V. (DIN)) 12,349, and the Privacy Impact Assessment according to CNIL/PIA were ensured.

## Results

Primarily, the results of the performance assessment indicated by the users’ feedback were positive. However, physicians highlighted some issues with wearing HL2^TM^. Noticeable feelings of discomfort were experienced and the physician’s entire field of vision was obstructed by the visor, resulting in the loss of eye-to-eye contact with the patient which is an integral part of the examination. Therefore, HL2^TM^ was considered to be unsuitable for the ARMED use case. In contrast, the RWN500 was reported to be lightweight and comfortable to use. It superimposes information of the real world on the user’s view without blocking the user’s vision. Such criterion was among the frequent advantages in favor for RWN500 stated by the users during the test phase. Nevertheless, the display is small and requires some time to get used to. The intuitive form of voice-controlled RWN 500 facilitates work well and the active noise cancellation allows for precise voice control.

To ensure proper forensic photo documentation, it is crucial to ensure high-resolution so that even the smallest findings (< 0·5 mm) are displayed sharply in photos without smoothing artifacts. The display on the screen and/or the photos must be true to color. Conclusively, the quality of the camera of the HMDs is essential. The 48 MP camera of RWN500 met the criteria, whereas the 8 MP camera of HL2^TM^ did not yield the necessary results. However, photos taken with the RWN500 from distances either longer or shorter than 20 cm were also deemed inadequate for a thorough forensic assessment. The lack of image quality of the HL2^TM^ among other issues (Table 3) led to the decision not to pursue an AR/MR enhanced workflow, but instead, to establish an easy and reliable “workhorse” system. Consequently, AR/MR-related system requirements such as graphical user interfaces (GUIs) were not accomplished in this phase and postponed for later implementation of an AR/MR workflow if possible. Table 3 summarizes the advantages and disadvantages of both devices according to technical performance, simplicity of handling, and users feedback given during the pilot phase.

Regarding the quality of the ARMED video stream transmission, the direct examination of the injuries revealed equal results regarding the documented type, size, and shape when compared with the assessment via the ARMED video stream. However, the direct assessment was superior in the description of the color and boundary zone of the injuries.

ARMED has been successfully integrated at three partner hospitals: Ravensburg, Offenburg and Heilbronn. The hospitals in Ravensburg and Heilbronn have implemented ARMED into regular daily practice to be used when a case of violence is suspected.

**Table 3:** Advantages and disadvantages of the two tested HMDs used in the pilot phase

	Advantages	Disadvantages
Microsoft HL2^TM^	• Full AR/MR capabilities for comprehensive extended reality experiences. • Immersive holographic display. • Familiar Windows OS for user convenience. • Multitasking during video conferencing.	• Requires training for proficient use. • Hand movements and/or wearing examination gloves might be misinterpreted as controls. • Relatively heavy weight causes discomfort in extended use. • Susceptible to disinfectants, needing specialized cleaning (Clean Box). • Limited 2-3-hour battery life • Non-replaceable battery. • Additional headset needed for clear sound. • No direct eye contact between physician and patient.
RWN500	• User-friendly without extensive training. • Excellent voice control. • Robust, lightweight design. • 6–8-hour active battery life with Sphere©. • Main part disinfectable with common agents. • Washable, detachable headband. • High-performance 48 MP camera, even in low light. • Additional flash and steady lighting. • Advanced zoom with maintained image quality. • Effective video stabilization. • High contrast display for indoor/outdoor use. • Adjustable display position. • Efficient noise cancellation. • Direct eye contact between physician and patient.	• No AR/MR functionality. • Small display limits detailed information. • Relies solely on voice control.

## Discussion

The international agreement known as the Istanbul Convention introduced legal frameworks that obligate states to provide forensic examinations following incidents of violence, ensuring meticulous evidence collection and documentation. However, the implementation of such regulations in many countries, including Germany, remains unsatisfactory. Despite legal provisions in the German Social Code, many regions lack the capacity to offer forensic examinations, especially for cases unreported to the police. Shortage of forensic expertise and their limited mobility create a gap in addressing these cases, which involve sexual assaults, domestic violence, and child abuse.

When suspicious cases of violence arise and no forensic expert is available on-site, remote assistance becomes an efficient and fast solution without having to wait for an expert to arrive and provide help. In response, we developed the ARMED teleforensic platform as a practical, user-centered, and comfortable telemedicine solution to provide access to forensic expertise to empower physicians to perform examinations of victims of violence at an appropriate level of quality under forensic guidance. Therefore, ARMED facilitates a more comprehensive adherence to the provisions set forth in the Istanbul Convention and the German Social Code to support victims of violence, ensuring that both medical and legal aspects are appropriately addressed.

Telemedicine has become established in the WHO European Region in recent years; however, current challenges include user, technology, and infrastructure barriers. However, technological healthcare provision yields improved patient outcomes, enhanced follow-up by professionals, and logistical benefits [23].

Initially, it was planned to use an AR/MR solution, enabling physicians to access additional information through HMD. HL2^TM^ was thus chosen because of its capability to display holographically superimposed objects on the field of vision, which allow wearers to visualize written or drawn instructions from experts and incorporate visual aids such as a body diagram to pinpoint injuries [22]. During our tests it turned out that HL2^TM^ did not meet our criteria, primarily due to insufficient stream and weak image quality as well as various technical issues related to its usability which were also confirmed in other settings [24]. Consequently, we decided to defer the implementation of the AR/MR approach to a later stage, pending the availability of suitable hardware and concentrated on a solution using the lighter and more intuitively useable RWN500 providing assisted Reality (aR). Although aR may sound less glamorous than AR and MR, in the current use case, it proves to be a practical and accessible remote access technology. The use of professional camera is still considered an option whenever essential.

## Limitations

During the development of ARMED we encountered several limitations. Firstly, we had to modify the original AR/MR design to a simpler solution. The pursuit of a seamless examination environment, user-friendly devices, and maximum benefit for the victims prompted us to choose a non-augmented solution. Secondly, a major limitation was the availability of a stable internet connection with sufficient speed, which is not widely accessible. Broadband expansion in Germany is progressing slowly, and the technical infrastructure in hospitals requires significant improvement. This issue could cause problems when an ARMED examination is planned to be conducted in a location that is not already equipped for telemedicine. However, using location-independent network providers might offer a solution to this problem. Another limitation is that since ARMED has been implemented for routine use, two cases have been reported and examined via this system so far. Consequently, there is not enough available data to compare it to the conventional standard forensic examination to date. Hence, its effectiveness in real-world scenarios and its acceptance in legal proceedings remain to be studied. Further studies in this context will help close the knowledge gaps.

### Future plan

The next steps for effective implementation are profiling real cases using ARMED - in comparison to the conventional method - and validating the entire process from registration to submission to expert opinions and court proceedings. On the organizational level, our focus is expanding ARMED to include more hospitals across the country. Our medium-term objective is to ensure that victims of violence across the federal state of Baden-Wuerttemberg can access expert help within an hour. To achieve this goal, establishing individual agreements with external partner hospitals is necessary, as well as fostering collaboration with colleagues from forensic medicine institutes in other cities. This approach will help in creating a nationwide network of forensic experts who can provide their expertise to partner hospitals through ARMED, irrespective of time and location. When ARMED proves its effectiveness, its applicability to countries without any medical forensic care for victims of violence would be one of the greatest achievements. On the developmental front, the involvement of additional specialists, such as radiologists and psychiatrists and/or psychologists, is also crucial to enhance the system’s capabilities. Lastly, at the technical level, we are waiting for suitable hardware to become available to continue our research towards the implementation of XR/AR/VR.

## Conclusion

To bridge the gap of a limited number of forensic institutes and experts, ARMED creates an option for synchronous examinations of victims of violence even in currently underserved regions through remote involvement of forensic experts. It alleviates the burden on healthcare providers to enhance the quality of findings collection and evidence preservation as required by law. The ultimate goal is to improve the likelihood of appropriate medical, legal, and social outcomes in cases of violence and abuse.

## Data Availability

The data generated, analyzed, and/or evaluated during the development phases of the ARMED system are included in this published article. Additionally, any further inquiries regarding the development of the telemedical model ‘ARMED’ can be directed to the corresponding author, Y.M.T., upon request.

## Abbreviations

AI: Artificial Intelligence
aR: assisted Reality
AR: Augmented Reality
ARMED: Augmented Reality Assisted, Medical Evidence Collection and Documentation
CNIL: Commission Nationale de l’Informatique et des Libertés
DIN: Deutsches Institut für Normung e.V.= German Institute for Standardisation
DSFA: Datenschutz-Folgenabschätzung = Data Protection Impact Assessment
DSGVO: Datenschutzgrundverordnung = General Data Protection Regulation
GDPR: General Data Protection Regulation
HL2^TM^: HoloLens2^TM^
HMD: Head-Mounted Display
GUIs: Intuitive Graphical user interfaces
ID: Identification
IFTM: Institute for Forensic and Traffic Medicine
LAN: Local Area Network
PACS: Picture Archiving Communication System
PC: Personal Computer
PIA: Privacy Impact Assessment
RWN500: RealWear Navigator 500
SGB: Strafgesetzbuch = Criminal Code
UKHD: Universitätsklinikum Heidelberg = Heidelberg University Hospital
VR: Virtual Reality
WLAN: Wireless Local Area Network
WHO: World Health Organization
XR: Extended Reality
